# History of a Case of Pneumothorax, with Recovery

**Published:** 1856-11

**Authors:** J. Forsyth Meigs


### MEDICAL EXAMINER.

NEW SERIES.—NO. CXLIII.—NOVEMBER, 1856.

---

### ORIGINAL COMMUNICATIONS.

---

### *History of a Case of Pneumothorax, with Recovery.*

By J. Forsyth Meigs, M. D.

S. G., a boy, eleven years of age, passed the summer of 1846 on the eastern shore of Maryland. In July he was seized with a violent bilious fever, in one of the paroxysms of which he had a slight convulsion, followed by comatose symptoms. He recovered partially, and was removed to Philadelphia, where I attended him during the months of September and October with obstinate intermittent fever, the paroxysms being irregular in their periodicity, and the intermissions often very imperfect. He rarely had distinct chills, but would have instead a disposition to collapse, and sometimes merely violent pains in the limbs. Quinine failed to relieve him, as did also the port wine and red bark mixture. He finally recovered under the use of large doses of citrate of iron and quinine.

*Dec. 17th.* He had last evening a return of his fever, it being just twenty-eight days since his last paroxysm. This morning he was running about again, but at 12 M. complained of pain in the joints, when he went to bed and was soon seized with violent fever. When I saw him in the afternoon the pulse was 120, the skin very hot and dry, and the face colored of a deep mahogany red tint; he was stupid, so that it was difficult to obtain answers from him, and he had severe headache. He has had some cough for three days past. Ordered 30 leeches to the temples; calomel, 4 grains, in syrup of rhubarb, to be followed by syrup of rhubarb again in four hours, if he have no stool; cold to the head, and a mustard foot-bath. In the evening his condition was the same, and he was ordered an enema.

*Dec.* 18*th.* At 2½ A. M. he had a slight convulsion, with clonic movements of the limbs and extasis. He was bled to six ounces, and another enema was ordered. He continued very sick throughout the day, with some cough, and complains of pain in right side just below the axilla. The spleen was tumefied; no enlargement of the liver could be detected; respiration 28; pulse 120. Neutral mixture ordered.

*Dec.* 19*th.* Imperfect remission in the morning, when 3 grs. of quinine every hour were prescribed. He took 15 grs. when the fever returned and the quinine was suspended.

In the evening the respiration was frequent and laborious, and he had severe stitch in the right side, after coughing or breathing deeply. The skin was hot and dry, and there was a brick-dust flush upon the cheeks. Dr. C. D. Meigs now saw him with me. A blister was applied beneath and back of the right nipple, and one of the following powders ordered to be given every two hours: ℞. Hydrarg. chlor. mit., gr.vi; Antimon. sulphuret. præcip. gr. iii.; Potass. nitrat. gr. xii. In chart. no. xii. He was to take an effervescing draught intermediate to the powders.

*Dec.* 20*th.* Very sick. Respiration 40; pulse 120; cough frequent and painful. To be dry cupped over the back.

*Dec.* 21*st*, 22*d*, 23*d*. No change, with the exception of a slight remission without perspiration in the morning. Throughout the day, and early part of the night, the fever was violent, the face deeply flushed, and he had severe pain and cough. There was complete anorexia. On the 21st the calomel was left out of the powders, and on the evening of the 23d, as he was very weak, the antimony and nitre were suspended, and he was ordered one grain of Dover's powder every few hours, as might be necessary, and a tablespoonful of wine whey every two hours.

*Dec.* 24*th.* Face hippocratic; very drowsy; pulse 132, and respiration 52. Complains very much of pain affecting the whole right side of the chest, which he says also is very sore. The stitch during coughing and breathing continues. The cough is very frequent, but he has not expectorated at all, though sometimes after coughing severely he seems to bring phlegm into the throat and swallow it.

I have examined the chest repeatedly since the beginning of the attack, but have never been able to satisfy myself of the existence of pneumonia, though he has had all the rational signs of the disease, except the expectoration. The only physical sign he has presented has been rudeness of respiration over the lower third of the right side behind, but there has been neither bronchial respiration, crepitant, nor sub-crepitant râle.

This morning, on percussing the chest in front, I was amazed to find the whole of the right side loudly tympanitic. On the left, the percussion was natural. On auscultation, there was heard over the right side a feeble respiratory murmur, obscured by a loud amphoric respiration. There was scarcely any movement during respiration over the right side of the chest. It was impossible to examine the chest behind, because of the weakness of the patient.

Throughout the day the patient continued very weak. The skin was hot and dry; the tongue covered with a thick dirty fur, and dryish; the cheeks presented each a distinctly circumscribed dark red flush, whilst the forehead was white; the eyes were dull, and he was very drowsy, but perfectly intelligent when roused.

Ordered in the morning a tablespoonful of wine whey every hour; also small quantities of ice cream, calves' feet jelly and partridge tea, to be given frequently as doses; all medicine except the Dover's powder to be suspended.

*Dec. 25th.* A restless night, with constant fever. This morning the respiration is 42, and the pulse 126; the skin is hot and dry, the tongue much coated, and the countenance rather better. The cough is rather frequent, but less painful than before. There is no headache; he is drowsy, but intelligent when roused. No stool; abdomen slightly tympanitic; physical signs as before.

In the afternoon and evening the patient was more comfortable. He had less pain and coughed less; there was some perspiration, and the tongue became moist. He has taken three powders through the day.

*Dec. 26th.* Has had a comfortable night, and took this morning some food with pleasure, for the first time. He has had two stools. The face is pale, the countenance a good deal sunken, and the eyes dull. The hands are sallow and shrivelled; the tongue dry and coated; the skin only slightly warmer than natural; the pulse 128, and of moderate volume; and the respiration 40, even, and not laborious; he has no pain except after violent cough, and the cough is less frequent. The percussion is less sonorous, and the chest movement on the right side more considerable than at the last examination. The powders to be suspended; wine whey continued, and chicken water, and bread and milk allowed for diet.

*Dec. 27th.* From midnight to 2 A. M. he had a slight collapse. He was very pale, and looked ill; one cheek was cold, whilst the other was hot and flushed; he had cold hands and feet; the respiration was 30; and the pulse small and feeble. Under increased doses of wine whey, and warm coverings, he reacted, and at 9 A. M. the skin had become slightly warmer than natural, and dry, and the hands were hot and parched. The respiration was 40, even, and with moderate effort; the pulse was 100, of moderate volume, and not weak; the tongue moist, reddish, and clean in patches; has had one stool; one cheek was flushed, and the other pale. The intelligence was perfect, and there was no unusual drowsiness. He does not cough much, and has no pain except after a deep hard cough. To take two grains of quinine every hour.

In the evening the pulse had fallen to 84, and the respiration to 32; the skin was of the natural temperature, soft, and moist, and there had been free sweating through the afternoon. Has taken five doses of the quinine. To take two more of the quinine powders, if no fever appear, and to take a grain of Dover's powder if necessary.

*Dec. 28th.* Has taken two more doses of quinine, and one Dover's powder. At midnight he had another cold fit, marked by cold hands and feet; pinched face; cold and clammy sweat; and feeble pulse. This morning the respiration is 22, even, silent, and easy; the pulse is 72, rather small, and compressible; the skin naturally warm, soft, and moist; the tongue is cleaner, and moist; he coughs but little, and has no pain. To take weak milk punch, with bread and milk.

*Dec. 29th.* Doing well. Respiration 30; pulse 75; tongue clean and moist; he desires food, and is to take a few roasted oysters. He is in excellent spirits, smiling and talking quite cheerfully. Coughs rather more than yesterday, and has ejected a few thick mucous sputa. To take four doses of quinine to-day.

Percussion on the right side, over and for an inch beneath the clavicle, is but little more sonorous than on the left From this point down to the liver it becomes highly sonorous, almost tympanitic indeed, but much less so than a few days back. In the infra-clavicular region, the respiration is vesicular, but puerile, and attended with a loud prolonged expiratory sound. Beneath this it is loudly amphoric, the expiration being as distinct and long as the inspiration. Over the upper third of the chest is heard through the amphoric sound, a well marked sibilant, but no sub-crepitant râle. In the same region is heard, in connexion with the amphoric inspiration and expiration, a loud sound, which appears to be a bruit de frottement of a coarse and harsh character. There is no metallic tinkling either in the cough or voice.

The respiratory sound over the left side is natural, but exaggerated.

*Dec. 30th.* No fever; pulse 78; respiration 28; cough slight and he has no pain; appetite moderate; the physical signs are rapidly improving; percussion yields a nearly natural sound from above, to midway between the clavicle and nipple, while below the nipple it is still very sonorous. Over a space of two of three inches below the clavicle there is a perfectly natural vesicular respiration, except that expiration is still too long and loud. The inspiration is long, soft, and gradual; the expiration short, but rude. Over the mammary region and below it, vesicular murmur is audible, but it is much less perfect than above, and is mingled with a kind of tubal blowing in both times.

I endeavored to examine the back, as I had attempted before, but the patient was so weak and so irritable as to render it impossible.

The quinine is continued, three or four of the powders to be taken every day until suspended. Diet light and nutritious.

*January 3d*, 1847. Improving rapidly. Is now able to walk across the room; sits up a good deal and looks cheerful. Respiration 22; pulse 68. On examining the chest I found the percussion natural on the right side in front, with the exception of a rather greater sonoriety than usual near the nipple. The respiratory sounds are natural in front, except just over the mammary region, where they are a little rude, and attended with an expiratory murmur. Behind, in the right side, the percussion is duller at the lower margin of the chest than above, and the vesicular murmur is weak, but quite audible.

The right side is somewhat contracted; the right shoulder falls and the intercostal spaces of that side are more marked, more sunken, and narrower.

*January* 17*th.* Getting on very well; has been down stairs and has a fine appetite, and has gained flesh and color; he has no pain, except sometimes a little when he gapes; no cough whatever. On examining the chest I found a healthy percussion and vesicular murmur over the upper third of the right side, both before and behind. The lower two-thirds presented a rather duller sound on percussion, and a somewhat feebler vesicular murmur on the right than on the left side, both before and behind. The right side was slightly contracted, and the right shoulder a little fallen.

*January* 6*th*, 1850. I have seen the subject of this case to-day. He is in fine health, having grown tall, stout, and muscular. He shows no sign of pulmonary disease. Percussion yields a sonorous sound over the whole dorsal region, with the exception of very slight dulness over the lower third of the right side. Respiration strong and distinct over the whole back.

I have seen my patient again this year (1856), and find him grown into a hearty, active man, without any appearance of feeble health. He has never had any trouble whatever with his chest since his recovery.

*Remarks.*—It is to be regretted that the details of the above case, in regard to the physical signs, are not more full. There were two causes for this: one was my own want of a better habit of observation at the time of its occurrence, and the other was the extreme severity of the illness, which made it impossible to examine the child in a satisfactory manner. To these were added the difficulties which always attend upon cases in private practice. In spite of this defect, however, it is a highly interesting case, and one quite worthy of being put on record. The existence of pneumothorax is incontestable, and so also is the fact that the pneumothorax was the result of an acute pulmonary inflammation. Moreover, the original pulmonary inflammation was complicated with severe intermittent fever. The recovery of the patient under such adverse circumstances adds another to the numerous examples of the wonderful beneficence of nature's efforts to preserve life under the most alarming condition of disease. To myself, the recovery of this patient has still additional interest: it shows how much the prognosis of the physician ought to be based upon the constitution of the patient. The family to which this child belongs is of remarkable vigor, and has afforded me two other instances of recovery from most dangerous attacks of disease.

Two cousins of this boy, a brother and sister, recovered from severe membranous croup. One, the girl, exhibited the most surprising escape from illness that I have ever seen. The disease not only attacked the larynx, but, after several days of great severity, reached such a height as to cause, on two occasions, absolute suspension of animation, marked by total insensibility, falling of the jaw, cessation of the pulse at the wrist and elbow, and, for a few moments, apparent death.

The most frequent cause of pneumothorax, both in adults and children, is well known to be the rupture of a tubercular cavity into the pleural sac. The correctness of this opinion is proved by M. Saussier (Comp. de Méd. Prat., t. vii, p. 135.) Of 131 cases of pneumothorax, he found the cause of that affection to be phthisis in 81, pleurisy in 29, gangrene of the lung in 7, emphysema in 5, pneumonic abscess only in 1, and other morbid conditions in the remainder. The following facts show the same thing to be true of children: Of 21 cases occurring in children, (11 from MM. Rilliet and Barthez, 6 from M. Barrier, 2 from M. Constant, 1 from M. Berton, and 1 from M. Fauvel,) 15 were shown to be the result of tubercular disease, 5 of pneumonia, and one of gangrene of the lung. In the case detailed above, however, there was no ground whatever for suspecting the presence of tubercles in the lungs, and it is necessary therefore to explain the production of the pneumothorax in some other mode. My own opinion is that it was clearly the result of the rupture of a small pneumonic abscess which had formed during the progress of an attack of pleuropneumonia. My opinion is based upon the following considerations.

The contraction of the chest which followed the recovery, with the character of the symptoms, both physical and rational, during the progress of the case, are sufficient proof of the existence of pleurisy. And yet the case was not one of empyema, for the amount of effusion was small, nor was there, at the moment when the pneumothorax showed itself, any expectoration of pus in masses, as there should have been, had an empyema suddenly found its way into a bronchus. The absence of any large pleuritic effusion is shown by the limited degree of dulness on percussion, and by the fact that the respiratory sounds were not suppressed over the lower parts of the thorax.

The existence of pneumonia appears probable from the severity and persistence of the febrile reaction, from the fact that the cough was at times loose, indicating the rejection of sputa into the fauces, though there was no evident expectoration, and from the fact that sibilant râle was heard during the recovery. Lastly, the difficulty of explaining the perforation of the lung in any other mode than by the rupture of a pneumonic abscess raises this probability almost to a certainty. That such rupture is a common cause of pneumothorax in children, is proved by the statistics given above, where it is shown that of 21 cases occurring in children, the effusion was caused by pneumonia in 5, which is a vastly larger proportion than in adults.

This case is very interesting also from the fact of the recovery. The prognosis of pneumothorax is held by all authorities to be most unfavorable. That such must be the case is evident from the very nature of the affection. The danger is two-fold: first, that arising from the pressure on the vital organs of the thorax of the large quantities of air poured out into the pleural sac; and second, that arising from the disease which has itself been the cause of the pneumothorax. The danger is greater in adults than in children. M. Saussier reports 16 recoveries out of 147 cases, most of which had no doubt occurred in adults. Of 22 cases in children collected by myself (21 from different authors, and my own case) 5 recovered. The proportion in M. Saussier's cases was 1 in 9, while in the latter series it was 1 in about 4½. The reason why a larger proportion of recoveries take place in children than in adults is, no doubt, the greater frequency in them of pneumonia as the cause of the pneumothorax. Another reason is, the greater recuperative energy of children, a feature so marked in early life as to have given to that period, the title of the age of resurrections. Of the 5 recoveries above noted, 3 occurred in 15 phthisical and 2 in 6 pneumonic patients. The recoveries from pneumothorax in phthisical patients refer, of course, only to the disappearance of the symptoms and effects caused by the gaseous effusion, the tubercular disease remaining, probably, to a great degree, unaffected by the accident of the pneumothorax.

